# Temporal and spatial dynamics of harmful algal bloom-associated microbial communities in eutrophic Clear Lake, California

**DOI:** 10.1128/aem.00011-25

**Published:** 2025-03-28

**Authors:** Isha Kalra, Brittany P. Stewart, Kyra M. Florea, Jayme Smith, Eric A. Webb, David A. Caron

**Affiliations:** 1 University of Southern California 5116 , Los Angeles, California, USA; 2 Southern California Coastal Water Research Project 271871 , Costa Mesa, California, USA; Georgia Institute of Technology, Atlanta, Georgia, USA

**Keywords:** harmful algal blooms, Clear Lake, *Microcystis*, *Cyanobium*, *Dolichospermum*, *Nodularia*, microcystins, heterotrophic bacteria, microbial eukaryotes

## Abstract

Clear Lake is a large, natural eutrophic lake located in northern California, United States. Persistent, toxic cyanobacterial blooms have been reported in the lake since 2011. However, our understanding of the spatiotemporal distribution of toxin-producing genera and their interaction with the biotic and abiotic environment is limited. Moreover, few studies have addressed how the co-occurring microbial communities respond to these toxic cyanobacterial blooms. Using multi-domain 16S/18S rRNA gene amplicon sequencing, a strong seasonal succession within the cyanobacterial and co-occurring eukaryotic assemblage was identified, which was primarily explained by variation in total phosphorus (~30%, *P* < 0.001) and temperature (~15%, *P* < 0.01). Cyanobacterial seasonal succession was often initiated by proliferation of diazotrophs (*Dolichospermum* and *Nodularia*) with concomitant increases in total nitrogen, followed by blooms of non-diazotrophs, such as *Microcystis, Limnothrix,* and *Planktothrix*. The picocyanobacterium *Cyanobium*, previously undocumented in the lake, was a dominant summer taxon in the western part of Clear Lake, accounting for ~45%–80% relative abundance of the cyanobacterial reads. Seasonal succession within the eukaryotic assemblage was influenced by photosynthetic chlorophytes and diatoms, as well as mixotrophic ciliates and cryptophytes. Among all toxin-producing cyanobacterial genera, *Microcystis* abundance was most strongly correlated with microcystin concentrations (*P* < 0.001), both of which appeared to influence co-occurring eukaryotes. Finally, using putative relationships based on correlation of sequence abundance and environmental variables, several potential grazers of *Microcystis* were identified, including cyclopoid copepods and *Cryptomonas*. These correlations need further confirmation and experimental work to validate the nature of the relationships.

**IMPORTANCE:**

Clear Lake is an important habitat for fish and wildlife, which also provides a myriad of human benefits, such as recreation, irrigation, and drinking water. Moreover, the lake is vital for tribal tradition and cultural practices. However, since the last decade, the lake has experienced recurring harmful algal blooms with toxin levels that frequently exceed California voluntary guidance levels. These high toxin concentrations pose a substantial threat to the residents, visitors, and tribal sustenance fishing and beneficial uses. However, significant gaps remain in our understanding of these toxic algal bloom dynamics and their interaction with the abiotic and biotic environments. This study characterized the seasonal and spatial patterns in the distribution of bloom-causing cyanobacteria and identified *Microcystis* as the major toxin producer in Clear Lake. Additionally, the co-occurring bacterial and eukaryotic microbial communities were also characterized, and their potential interactions with the cyanobacterial assemblage were identified and discussed.

## INTRODUCTION

Clear Lake is one of the oldest lakes in North America (approx. 2.5 million years old) as well as the largest natural lake completely within the state of California (177.2 km^2^) (1). The lake has a unique morphology and is divided into three main basins which often behave as separate entities; the largest basin of Upper Arm to the west (127 km^2^), followed by Oaks Arm (37.2 km^2^) to the northeast and Lower Arm (12.5 km^2^) to the southeast. Overall, the lake is warm and shallow, with maximum depths of approximately 18 m in the Oaks and Lower Arms, and 12 m in the Upper Arm (1). The large surface area, geographic setting at ≈430 m altitude, and shallow depth of the lake contribute to frequent water column mixing, making it a polymictic lake throughout the year (1). As a naturally eutrophic lake, Clear Lake has been a historically productive environment with early documentation of large phytoplankton biomass and high levels of primary productivity (2). Human activities in the past few centuries have contributed to anthropogenic nutrient loading in the system, resulting in the hypereutrophic status currently observed in the lake (3). Consequently, high densities of cyanobacterial blooms have been observed starting mid 1970s (1, 2). Cyanobacterial blooms in Clear Lake in recent years have been credited with decreased water quality, noxious odors, multiple cyanobacterial toxins (cyanotoxins), and other detrimental effects on human and wildlife uses of the lake.

Clear Lake has historically been considered nitrogen limited, and that element therefore has been thought to play an important role in determining phytoplankton community composition. Early studies reported that nitrogen fixation was responsible for more than 40% of total nitrogen flux into the lake (4). Consequently, nitrogen-fixing cyanobacteria have been assumed to have a competitive advantage over non-diazotrophs in Clear Lake (1, 5). In agreement with those findings, cyanobacterial assemblages in Clear Lake during the mid-1970s to 1990s were dominated by diazotrophic cyanobacteria such as *Anabaena* and *Aphanizomenon*, in addition to the non-diazotrophic cyanobacterium *Microcystis spp*. (1, 5). A change in the inorganic nitrogen chemistry of Clear Lake has also occurred over the last few decades (5). The dominant form of inorganic nitrogen has shifted from nitrite + nitrate to ammonia. These changes indicate that the microbial community of Clear Lake in recent decades is most likely supported through regenerated nitrogen via plankton excretion and microbial remineralization.

Contrary to nitrogen, the phosphorus status of Clear Lake has been assumed to be relatively replete with respect to phytoplankton growth requirements until recent decades (pre-2001) (3). This status has changed presumably as a consequence of natural and anthropogenically enhanced external nutrient loading of the lake, and perhaps due to changes in nitrogen availability as noted above. Additionally, phosphorus is a common target for reducing nutrient loading leading to algal/cyanobacterial blooms in fresh waters because there are no large, easily transformable pools of phosphorus, as there is for nitrogen (i.e., atmospheric nitrogen). To alleviate the effect of excess phosphorus on lake water quality resulting from cyanobacterial blooms, the total maximum daily loads (TMDLs) for phosphorus were implemented by the state of California in Clear Lake in 2007 (6) .

Despite these efforts to reduce phosphorus levels in Clear Lake, a recent review of long-term data from the lake (3) has revealed no significant reductions in phosphorus concentrations following the implementation of the TMDL, and the lake has experienced recurring harmful cyanobacterial blooms during the last decade (7). These persistent toxic cyanobacterial blooms have been especially problematic during the summer and fall seasons. During these months, microcystin concentrations have routinely exceeded caution (0.8 µg/L), warning (6 µg/L), and even danger (20 µg/L) levels based on the California voluntary guidance levels for the protection of human and animal health in recreational waters (8). For example, one major toxic event was recorded in the summer of 2016 when microcystin concentrations reached 16,920 µg/L in some areas of the lake (3). Furthermore, the cyanobacterial species dominating the bloom have varied in time and space in the lake. Due to the recurring bloom issues and the lake’s importance to tribal tradition and cultural uses, subsistence fishing, and recreation, several Clear Lake sites are routinely monitored by multiple Tribal and local Environmental Protection Departments for cyanotoxins, cyanobacterial species identifications and abundances, as well as other water quality parameters (https://www.bvrancheria.com/clearlakecyanotoxins).

Traditional, microscopy-based studies of the cyanobacterial assemblage in Clear Lake as well as a few molecular surveys have documented numerous genera present in the lake, several of which are known toxin producers (3, 7, 9). In addition to the previously mentioned *Anabaena*, *Aphanizomenon,* and *Microcystis*, other toxin-producing taxa, such as *Lyngbya*, *Dolichospermum,* and *Gloeotrichia*, have been frequently reported in Clear Lake since 2009 (3, 7, 9). Thus, Clear Lake suffers from multiple cyanobacterial harmful algal blooms (cyanoHABs), and recent surveys have demonstrated that both diazotrophic and non-diazotrophic taxa coexist in the lake.

While bottom-up factors, such as nutrient concentrations, temperature, pH, etc., significantly affect cyanoHAB proliferation, interactions with the co-occurring microbial community also play a substantial role in shaping the overall cyanobacterial assemblage and their toxicity (10). Specific groups of heterotrophic bacteria have been shown to be associated with toxic cyanobacterial taxa in natural communities (11) and may either help proliferate (12) or suppress their growth (13). In addition, changes in abundance and species composition of the eukaryotic algal assemblage (14, 15) as well as interactions with grazers (16) and fungi (17) also influence cyanobacterial bloom dynamics. Therefore, to fully understand the dynamics of toxic cyanobacterial blooms, changes associated with the co-occurring bacterial and eukaryotic assemblage should also be characterized.

Despite numerous biological surveys using traditional methodologies, a thorough molecular investigation of the microbial community of Clear Lake is still lacking. The specific taxa that are the cause of toxins (predominantly microcystins but also anatoxin-a) observed in the lake are not presently known nor is the effect that these species and/or cyanotoxins have on the species richness and community composition of the co-occurring microbial assemblages. Similarly, the relative importance of biotic and abiotic factors in controlling microbial community structure and cyanoHAB occurrence patterns is not fully understood. To address these questions, we employed ribosomal RNA gene sequencing of a region of the small subunit ribosomal genes (18S rRNA, 16S rRNA) to (i) provide extensive characterization of the species richness and community composition of the planktonic bacterial and microbial eukaryotic assemblage of the lake in the presence and absence of toxic cyanobacterial blooms; (ii). investigate the spatial and temporal (annual and seasonal) dynamics of the community; and (iii) evaluate the influence of environmental factors and cyanoHAB proliferation on microbial community structure.

## MATERIALS AND METHODS

### Study site and sampling scheme

**Fig 1:**
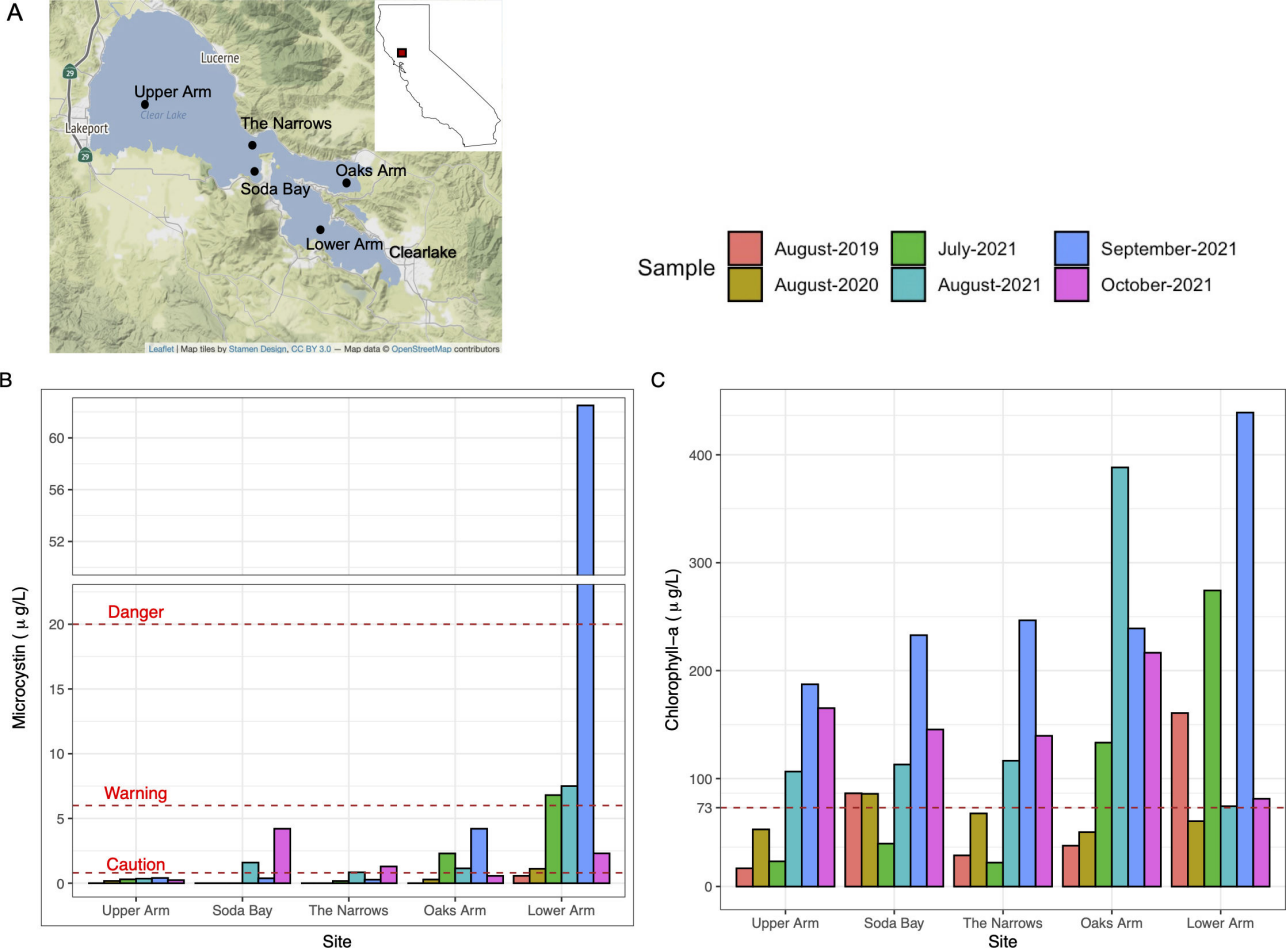
(A) Geographical map of Clear Lake. Sampling locations are indicated by black circles. Inset – location of Clear Lake in the state of California, United States. The map was created in R using the Leaflet package. (B) Microcystin concentrations at the sampling locations of Clear Lake during 2019, 2020, and 2021. California microcystin trigger levels are shown as dotted red lines (caution >0.8 µg/L, warning >6 µg/L, and danger >20 µg/L). (C) Chlorophyll *a* concentration at the five sampling locations of Clear Lake during 2019, 2020, and 2021. The TMDL target level for chlorophyll *a* (73 µg/L) is shown as red dotted line. Samples were collected during August 2019, August 2020, July 2021, August 2021, September 2021, and October 2021 as shown by different bar colors in B and C.

Sampling was carried out at multiple sampling sites in Clear Lake to characterize the interannual and monthly temporal variability (within the typical cyanobacterial growing season) of the microbial community because bloom intensity and dynamics are known to vary on both scales. Interannual variability was examined by sampling in August of three successive years (2019, 2020, 2021). Monthly shifts in species richness and community composition were evaluated across peak cyanoHAB season in four consecutive months during 2021 (July, August, September, and October). For each sampling campaign, samples were collected (in duplicates) away from the shore in the three major Arms of the lake – Upper Arm, Oaks Arm, and Lower Arm, and at two sites near the junction of these Arms (The Narrows and Soda Bay) to span the spatial breadth of Clear Lake and to evaluate spatial heterogeneity in the microbial community at the whole-lake scale (Fig. 1A).

### Sample collection

Clean, acid-washed polycarbonate water bottles were used to collect surface water samples at each site and were stored in dark coolers until processed at the field laboratory. For nucleic acid extraction, 100 mL of water sample was filtered onto 0.2 µm Supor filters (see below), flash frozen, and stored at −80°C until analyzed. In addition, 50 mL aliquots of whole, unfiltered water samples were collected for nutrient analyses and frozen until analyzed. Chlorophyll *a* samples were collected by filtering 25 mL of sample onto 25 mm GF/F filters and freezing until analysis. Microcystins were quantified from 90 mL of water collected in 120 mL amber bottles and frozen until analysis (see below). An RBR Concerto (RBR Ltd., Ottawa, ON, Canada) was deployed at all sites to collect additional physiochemical parameters including dissolved oxygen, conductivity, and temperature. Relative abundances of the dominant planktonic taxa at each sampling site and time were done on material collected using a 20 µm plankton net, preserved in 1% formalin, and stored at 4°C until analyzed. Samples were analyzed using dissecting and compound upright microscopy.

### Microcystin, chlorophyll-*a,* and nutrient analyses

The quantification of total microcystin concentrations was performed using ADDA ELISA test kits (Eurofins Abraxis, Inc. Warminster, PA, USA). This assay is designed to detect all microcystin and nodularin variants with the ADDA side group in bulk and does not provide data for specific congeners of the toxin class. Chlorophyll *a* filters were extracted in 100% acetone for 18–24 h in a freezer (−20°C), and analyzed fluorometrically (18). Duplicate filters were collected at all stations, and the average chlorophyll *a* concentration of the two filters was used for reporting.

Colorimetric nutrient analyses were conducted at Chesapeake Biological Laboratory (Nutrient Analytical Services, Solomons, MD) using EPA methods 365.1 and 353.2 to quantify total phosphorus and total nitrogen concentrations, respectively.

### DNA isolation and sequencing

DNA was extracted from duplicate Supor filters using the Qiagen DNEasy Power Biofilm Kit (Germantown, MD) for each sampling date and site. Cyanobacterial cells were lysed using five rapid freeze–thaw cycles after adding the first two solutions from the Biofilm Kit. Proteinase K was then added to further enhance lysing, and the samples were incubated for at least 4 h (max 12 h) at 55°C. The remaining DNA extraction steps were performed according to the manufacturer’s protocol.

16S and 18S rRNA gene amplicon libraries were prepared using 515F (5′- GTGYCAGCMGCCGCGGTAA −3′)−926R (5′- CCGYCAATTYMTTTRAGTTT – 3′) 3-domain primers (19) that amplify regions of both the prokaryotic and eukaryotic small subunit rRNA genes from the same DNA sample. Primers were obtained from Eurofins Genomics (Louisville, KY), and amplicon libraries were constructed according to Fuhrman Lab protocol (20). Resulting amplicon libraries were sequenced on Illumina Miseq 2 × 300 bp platform at USC Norris Comprehensive Cancer Center Molecular Genomics Core (Los Angeles, CA). Duplicate libraries were sequenced for each sample.

### Sequence analysis

The 16S/18S amplicon sequences were analyzed according to the Fuhrman Lab eASV pipeline for 515F-926R primer amplified microbial communities (21, 22; https://github.com/jcmcnch/eASV-pipeline-for-515Y-926R). As the total reads contained a mixture of 16S/18S amplicons, all sequences were separated into 16S and 18S rRNA gene amplicon reads through alignment to SILVA132 (23) and PR2 databases (24), respectively, using bbsplit (25). All sequences that aligned to the databases were then binned as either prokaryotic (16S rRNA) or eukaryotic (18S rRNA) reads. Subsequent analysis was performed separately for prokaryotic and eukaryotic sequences. Sequences of prokaryotic reads were quality trimmed, merged, and denoised using dada2, and the resulting amplicon sequence variants (ASVs) were classified using the sk-learn classifier trained on SILVA132 in QIIME2 (26). The NCBI nr/nt database was then used to assign taxonomy for some cyanobacterial ASVs that were not annotated with SILVA132. Sequences of eukaryotic reads were first quality trimmed and concatenated using bbduk. The concatenated sequences were then imported into QIIME2, denoised using DADA2, and the resulting ASVs were classified using the sk-learn classifier trained on the PR2 database.

A total of 11,256,658 raw reads were recovered from all the samples, which were then classified into 8,728 prokaryotic (16S) and 1,267 eukaryotic (18S) ASVs. Contaminant reads and ASVs were removed using the decontam (v 1.18.0) (27) and phyloseq (v 1.42.0) R packages. After decontamination, 10,853,875 (16S) and 346,868 (18S) reads remained, which corresponded to 8715 prokaryotic and 1,265 eukaryotic ASVs (Table S1). In addition, rare/low abundance ASVs (less than 10 reads) were also filtered prior to analysis. For diversity and statistical analysis, raw reads were normalized using total sum scaling to account for sample and sequencing depth bias. All data wrangling, analysis, and plotting were conducted in R (v 2022.12.0), and the associated scripts are available on GitHub (https://github.com/IKalra889/Clear-Lake-amplicon-manuscript).

### Statistical tests

All statistical tests were run using R packages. Beta-diversity (Bray–Curtis dissimilarity) calculation and PERMANOVA (analysis of variance) analysis were done using the adonis2 function in vegan (version 2.6-4). The R microeco v0.11.0 package (28) was used to conduct principal coordinate analysis (PCoA), redundancy analysis (RDA), and Spearman correlation analysis. Wherever required, the ANOVA test was used to calculate statistically significant group differences.

## RESULTS

### Physicochemical conditions associated with cyanobacterial blooms in Clear Lake

Microcystin concentrations in Clear Lake have regularly reached high levels for recreational water use in recent years, and Oaks and Lower Arms have been particularly problematic in this regard (3). Considerable temporal and spatial variations in microcystin concentrations were observed during our 3-year study (Fig. 1B; Fig. S1A and S2A), with microcystins detected at most sites and in most months during 2021. Consistently high concentrations were recorded in the Oaks and Lower Arms, with the highest microcystin concentration observed in Lower Arm in September 2021 (62.5 µg/L), where concentrations reached ~3 times the California recreational danger level (20 µg /L). Moreover, high variability in the detection and concentrations of microcystins was also observed during our 3-year sampling effort in August (Fig. 1B; Fig. S2A). Microcystins were consistently detected in Lower Arm during August of all three years but were significantly higher in 2021 relative to 2019 and 2020, with concentrations (7.5 µg/L) above warning level. Moreover, microcystins were prevalent throughout the lake in August and October 2021, with most sites exhibiting toxin concentrations near or above caution level during those months.

Like microcystins, chlorophyll *a* concentrations also exhibited wide seasonal, interannual, and spatial variability, with values ranging from 16.8 µg/L in Upper Arm during August 2019 to the highest concentration of 440 µg/L in Lower Arm during September 2021 (Fig. 1C; Fig. S1B and S2B). Overall, a positive albeit weak correlation was observed between chlorophyll *a* and microcystin concentrations. In our entire sample set, we found a significant positive correlation between microcystins and chlorophyll *a* concentrations (rho = 0.5487, *P* = 0.002) over the 3-year study period (Fig. S3). Moreover, chlorophyll *a* levels exceeded the TMDL threshold of 73 ug/L at several sites and dates during our 3-year study period (Fig. 1C, red dotted line). A site was defined as a “bloom” site if the chlorophyll *a* levels were at or above this threshold, and as a “non-bloom” site when the levels were below this threshold. In 2021, all five sites were in “bloom” state during the months of August, September, and October (Table S2). In July 2021, only the southeastern Oaks and Lower Arm sites were under “bloom” state. On the other hand, only one and two sites were under “bloom” state in August 2020 and August 2019, respectively.

Other environmental variables, such as total nitrogen concentration, total phosphorus concentration, and conductivity, also showed spatial and temporal variability (Fig. S1C, D, G and S2C, D, G). During 2021, total nitrogen and phosphorus showed significantly higher values during fall compared with summer months (Fig. S1C and D ). Moreover, significant annual differences were also observed. For example, compared with August 2019, August 2021 had higher TN and TP concentrations, as well as higher conductivity and temperature (Fig. S2C, D, E and G). As noted for chlorophyll and microcystin (see above), spatial variability was also observed in other environmental variables, as evident from the large inter-quartile ranges and outliers (Fig. S1 and S2). However, unlike microcystin and chlorophyll, which showed higher accumulation in the southeastern parts of the lake (Oaks and Lower Arms), no specific spatial trends were observed for other variables.

### Clear Lake bacterial assemblage composition and diversity

**Fig 2:**
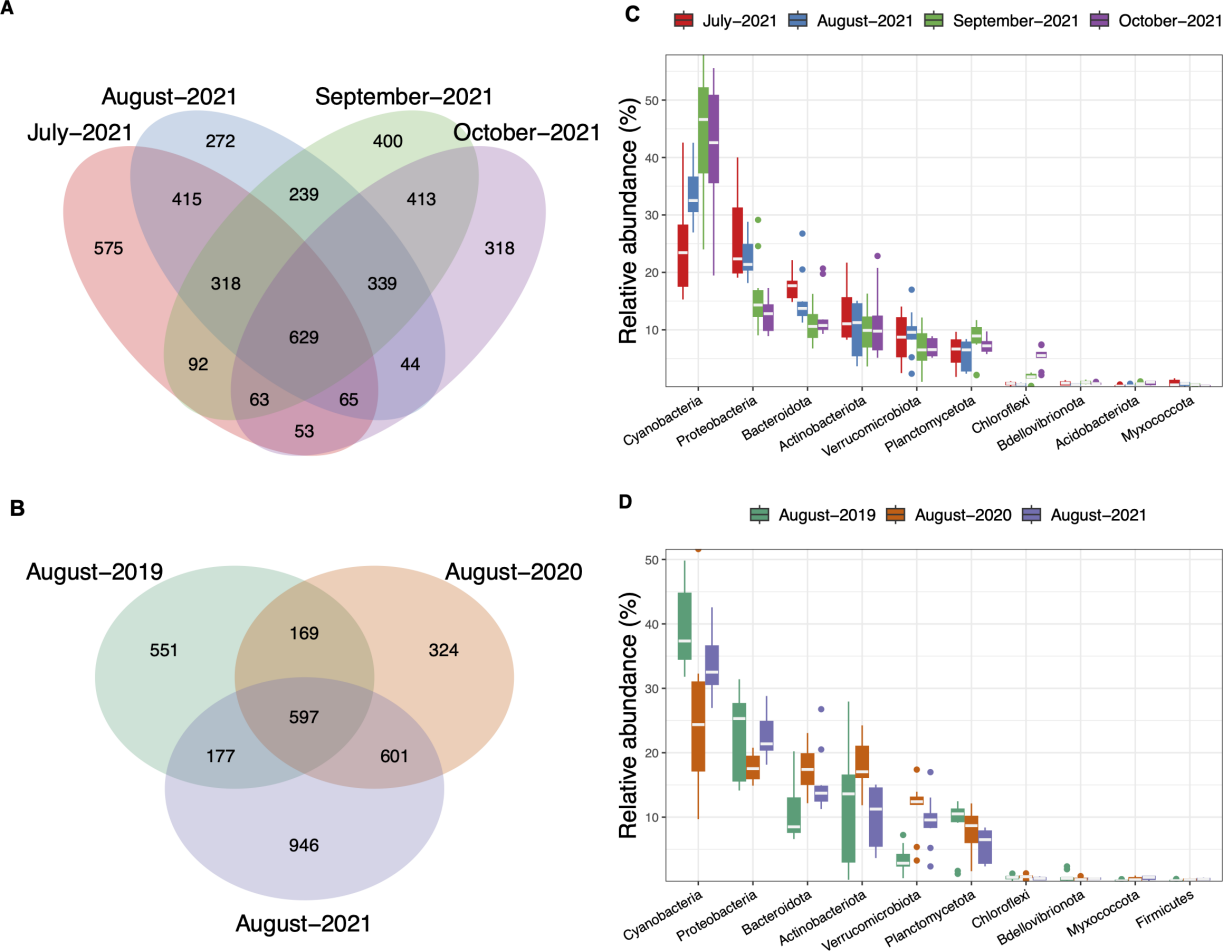
Bacterial assemblage diversity and composition in Clear Lake. Left: Venn diagrams depicting the number of unique and shared (intersections) ASVs for sample set collected during (**A**) summer and fall of 2021 and (**B**) August 2019, 2020, and 2021. Right: Relative abundance boxplots for the top 10 most abundant prokaryotic phyla found in (**C**) summer and fall of 2021 and (**D**) August of different years. In the boxplot, the horizontal line represents the median, the hinges represent the interquartile range (25%–75%) and the whiskers extend to 1.5xIQR. The points that fall outside 1.5xIQR are shown as outliers. Data are averaged for all the sites within the sampling date.

A total of 4,713 bacterial ASVs were observed in the total 60 samples analyzed after removing rare and contaminant reads. To understand seasonal and annual trends in occurrence of bacterial ASVs in Clear Lake, the number of unique and shared ASVs were calculated for four different months during 2021 (Fig. 2A) as well as August samples collected in three different years (Fig. 2B). The samples collected during summer and fall of 2021 revealed a “core” bacterial assemblage (i.e., ASVs shared by all samples during 2021) composed of 629 ASVs (Fig. 2A). Overall, cyanobacteria dominated the bacterial assemblage in Clear Lake; however, seasonal changes in the percentage contribution of individual bacterial phyla were also observed (Fig. 2C; Fig. S4A). Significantly higher percentages of cyanobacterial reads were observed during fall compared with summer, making up to 55%–57% of the total community. In contrast, the next most abundant phyla, Proteobacteria and Bacteroidota, were relatively more abundant in the summer compared with fall.

In our interannual survey, a total of 597 bacterial ASVs were shared among the samples collected in August in all 3 years (Fig. 2B). August 2021 samples had the highest number of unique ASVs and the highest Shannon diversity among all 2021 samples (Fig. S6A). The dominant bacterial phyla also followed similar trends in all 3 years, although some differences were observed between 2019 and 2021 (Fig. 2D). More significantly, large differences were observed among the samples collected on the same date at different locations in the lake, indicating site-specific differences in bacterial assemblage composition (note range of quartiles and whiskers in Fig. 2C and D; Fig. S4A and S5A).

### Clear Lake eukaryotic assemblage composition and diversity

**Fig 3:**
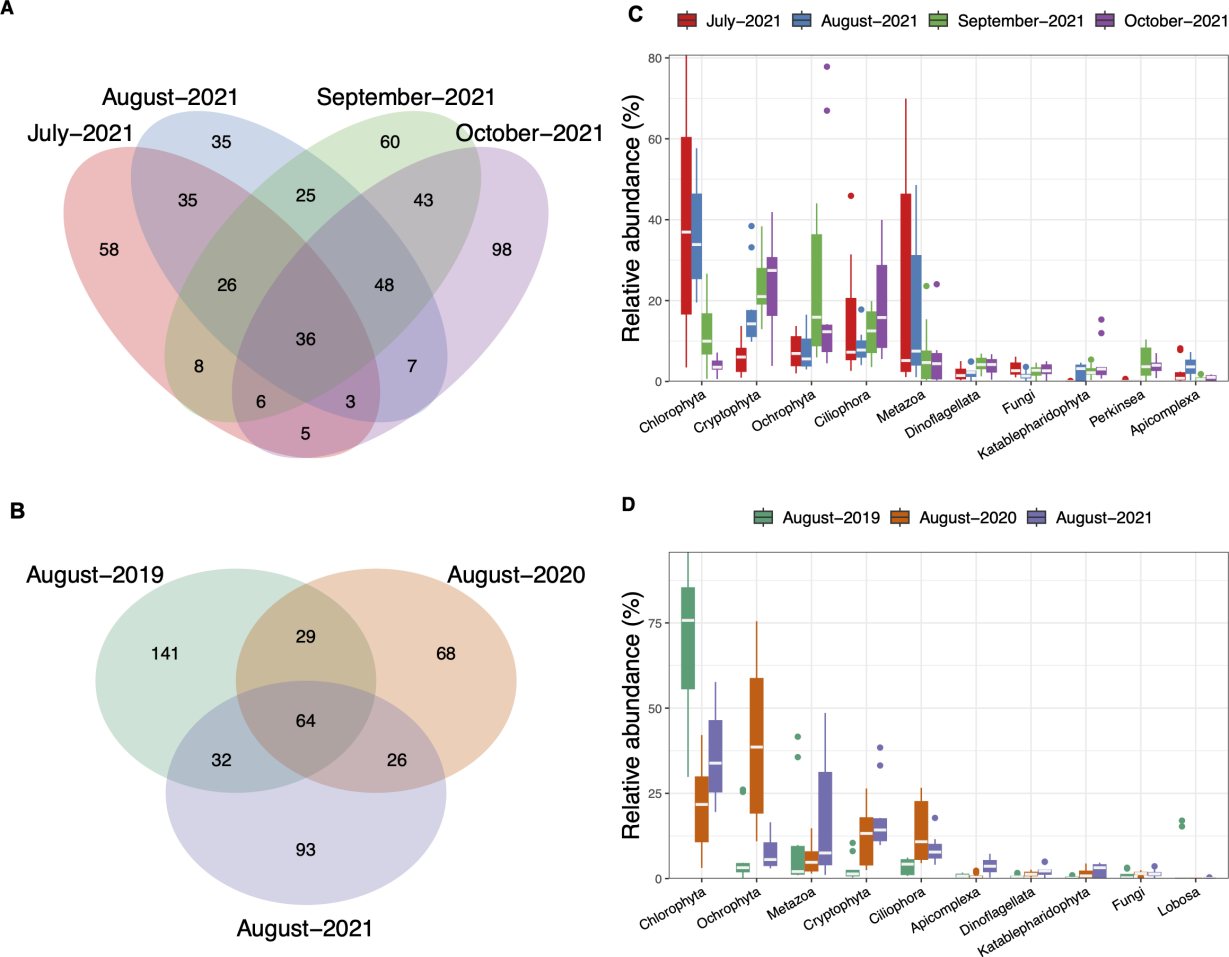
Eukaryotic assemblage diversity and composition in Clear Lake. Left: Venn diagrams depicting the number of unique and shared (intersections) ASVs for sample set collected during (**A**) summer and fall of 2021 and (**B**) August 2019, 2020, and 2021. Right: Relative abundance boxplots for the top 10 most abundant eukaryotic phyla found in (**C**) summer and fall of 2021 and (**D**) August of different years. In the boxplot, the horizontal line represents the median, the hinges represent the interquartile range (25%–75%) and the whiskers extend to 1.5xIQR. The points that fall outside 1.5xIQR are shown as outliers. Data are averaged for all the sites within the sampling date.

The Clear Lake eukaryotic assemblage was comprised of a total of 652 unique ASVs following removal of rare and contaminant ASVs. However, unlike the bacterial assemblage, only 36 eukaryotic ASVs were shared among the four monthly samples collected in 2021 (Fig. 3A). A relatively large percentage of the total number of ASVs observed (approximately half) was unique to each sampling month in 2021. The dominant eukaryotic taxa also varied for each month. For example, chlorophyte algae were highly abundant in July and August; however, cryptophytes and ochrophytes were more abundant during September and October (Fig. 3C; Fig. S4B). Additionally, heterotrophic groups, such as metazoans and ciliates, were also prominent members of the eukaryotic assemblage.

In our interannual survey of August samples, the “core” microbial eukaryotic assemblage (i.e., ASVs present in all 3 years) was comprised of 64 ASVs (Fig. 3B). Unlike the bacterial assemblage, however, the number of ASVs shared among all years was never greater than the numbers of ASVs that were unique to each year (141, 68, and 93 for 2019, 2020, and 2021, respectively). Moreover, like the bacterial assemblage, the proportion of the eukaryotic groups differed among the 3 years (Fig. 3D; Fig. S5B). For example, chlorophytes were especially dominant in August 2019 and 2021, constituting more than 75% of all reads (Fig. 3D), while ochrophytes dominated in 2020 (up to 75% of total reads) with chlorophytes second in abundance.

### Seasonal and spatial differences in the microbial communities of Clear Lake

**Fig 4:**
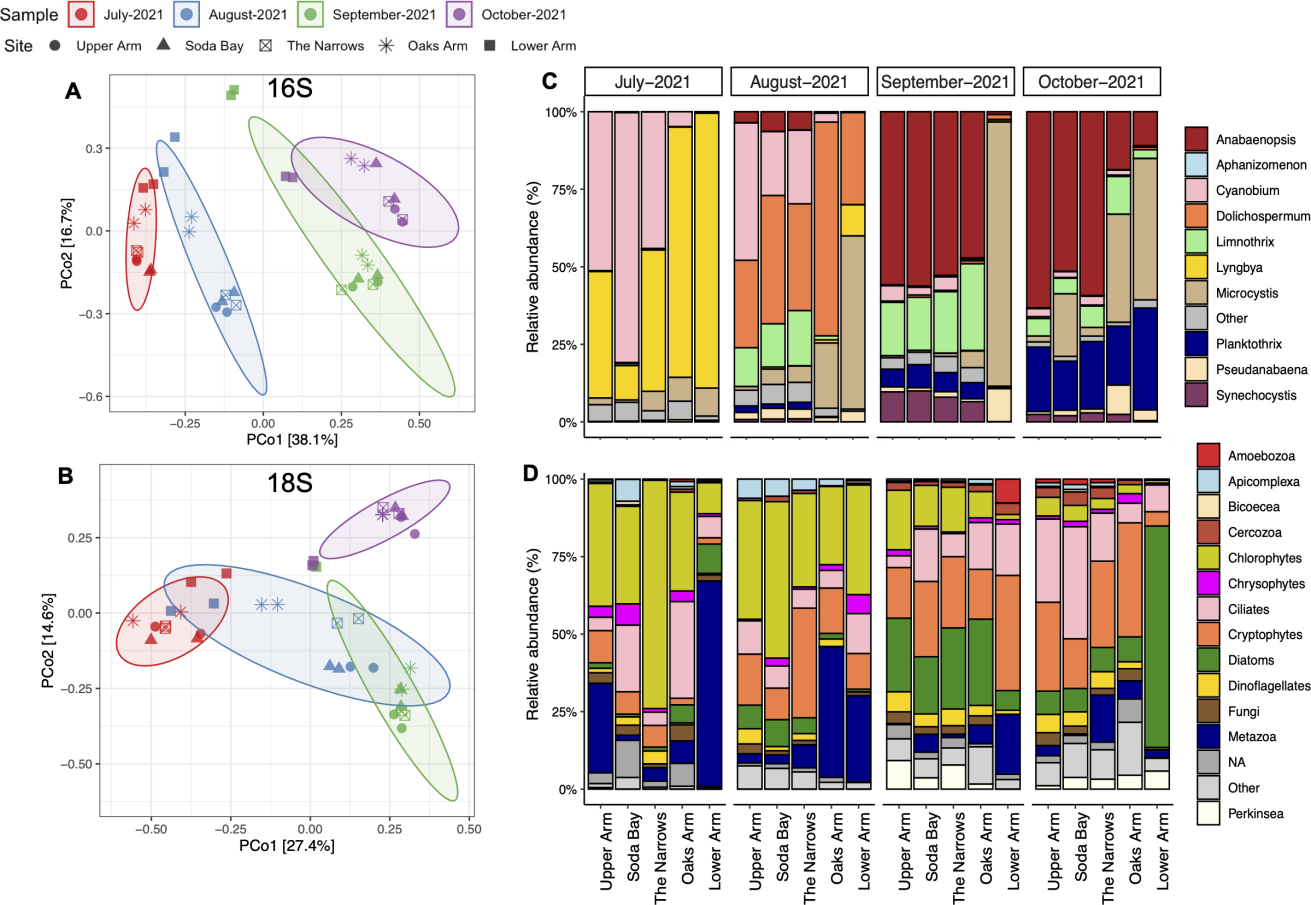
Monthly variations in microbial community diversity and composition at different sites in Clear Lake during summer and fall of 2021. PCoA ordination plots depicting Bray–Curtis dissimilarity of bacterial assemblage composition (**A**) and eukaryotic assemblage composition (**B**). Each point represents one sample or replicate. Sampling sites are depicted by different shapes, and the month of collection is represented by different colors (July–red, August–blue, September–green, and October–purple). Monthly changes in the relative abundance of major taxonomic groups of the cyanobacterial assemblage (**C**) at the genus level and eukaryotic assemblage composition (**D**) at the class or phylum level. Relative abundance data are averaged for the two replicates for each sample. The five sampling sites are shown on the *x*-axis.

**Fig 5:**
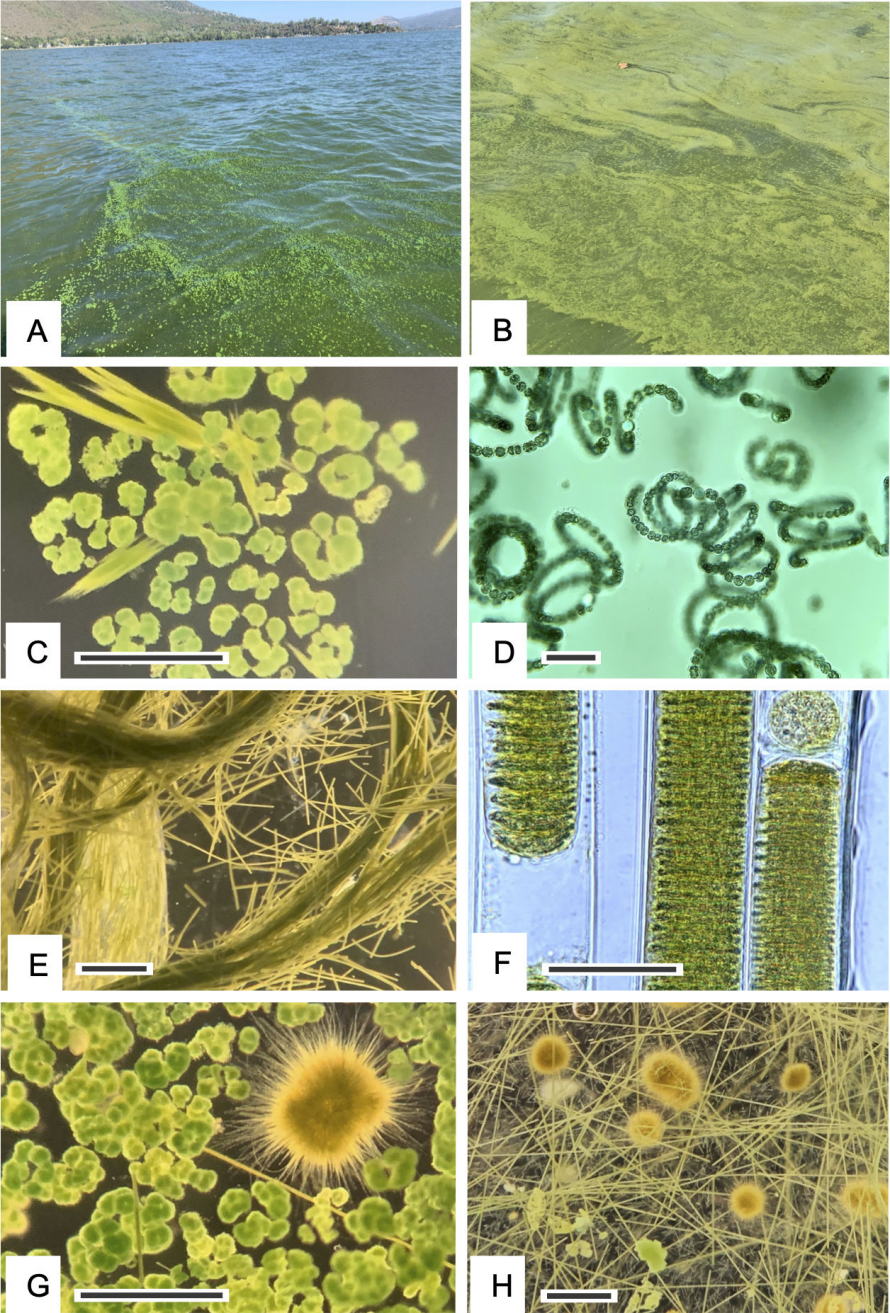
Representative images of surface manifestations of cyanobacterial blooms and the causative taxa observed in Clear Lake, CA during the study period (2019–2021). (**A**) Cyanobacteria accumulating at the surface of Konocti Bay, Clear Lake during the study, composed primarily of *Microcystis*. (**B**) Cyanobacteria accumulating at the surface of Honeymoon Bay, Clear Lake, composed primarily of *Lyngbya*. (**C–H**) Micrographs of various cyanobacterial genera commonly observed in Clear Lake during the study. (**C**) Dissecting photomicrograph of colonies of *Microcystis* (green clumps) and *Aphanozomenon* (filament bundles). (**D**) High magnification photomicrograph of colonies of *Dolichospermum*. (**E**) Dissecting photomicrograph of densely aggregated filaments of *Lyngbya*. (**F**) Individual filaments of *Lyngbya* at high magnification showing sheath surrounding filaments. (**G**) Dissecting photomicrograph of colonies of *Microcystis* (green clumps) and a colony of *Gloeotrichia* showing its radially arranged filaments. (**H**) A mixed assemblage of cyanobacteria including a few *Microcystis* colonies (small green clumps) and several colonies of *Gloeotrichia* (large yellow–brown colonies) with radially arranged filaments, among many dispersed individual *Lyngbya* filaments. Marker bars are 20 µm (**D, F**), 200 µm (**C, G**) and 500 µm (**E, H**).

Temporal and spatial differences in the bacterial and eukaryotic assemblages were evaluated on a monthly scale (July through October 2021) using Bray-Curtis dissimilarity and by comparing relative abundances of major taxa across the Clear Lake sites (Fig. 4). Principal coordinate analysis (PCoA) revealed that the bacterial assemblage aggregated by month and separated primarily along the first principal coordinate axis, explaining 38% of the variance in the distance matrix (Fig. 4A). This seasonal “succession” of the bacterial assemblage composition was evident from the differences in the relative abundances of major cyanobacterial and heterotrophic bacterial genera (Fig. 4C; Fig. S7). Particularly within the cyanobacterial assemblage, blooms of different genera often dominated in different months (Fig. 4C and 5). The dominant cyanobacterial taxon shifted from *Lyngbya* in July (75%–85% of cyanobacterial reads in Oaks and Lower Arms) to the diazotrophic *Dolichospermum* in August (25%–60% of reads) and then to the diazotrophic *Nodularia*/*Anabaenopsis* in September (Fig. 4C). These blooms of nitrogen fixers were followed by increases in the relative abundances of the non-diazotrophic *Microcystis*, which continued to dominate during the fall months, especially in the east part of the lake (Lower and Oaks Arms) (Fig. 4C; Fig. S7). Other non-diazotrophs, such as *Limnothrix* and *Planktothrix*, were also abundant during the fall months and appeared to follow *Nodularia* blooms. In addition to these bloom-causing taxa, the picoplanktonic cyanobacterium *Cyanobium*, previously undocumented from Clear Lake, was a dominant component of the summer cyanobacterial assemblage, specifically in the western part of the lake (45%–80% of cyanobacterial reads) (Fig. 4C).

Minor separation of the bacterial communities at each sampling site within each month was also observed along the second PCoA axis, which explained 16.7% of variance (Fig. 4A). In particular, Oaks and Lower Arm sites (asterisks and square symbols) clustered away from the other three sites. Moreover, although Upper Arm, Soda Bay, The Narrows, and Oaks Arm formed tight clusters within the 90% CI for a particular month, the composition of the Lower Arm sites was more similar across the summer and fall months. This clustering of Lower Arm samples across months could be explained by the dominance of *Microcystis* blooms (50%–90% of the cyanobacterial assemblage), especially during August, September, and October (Fig. 4C).

**Fig 6:**
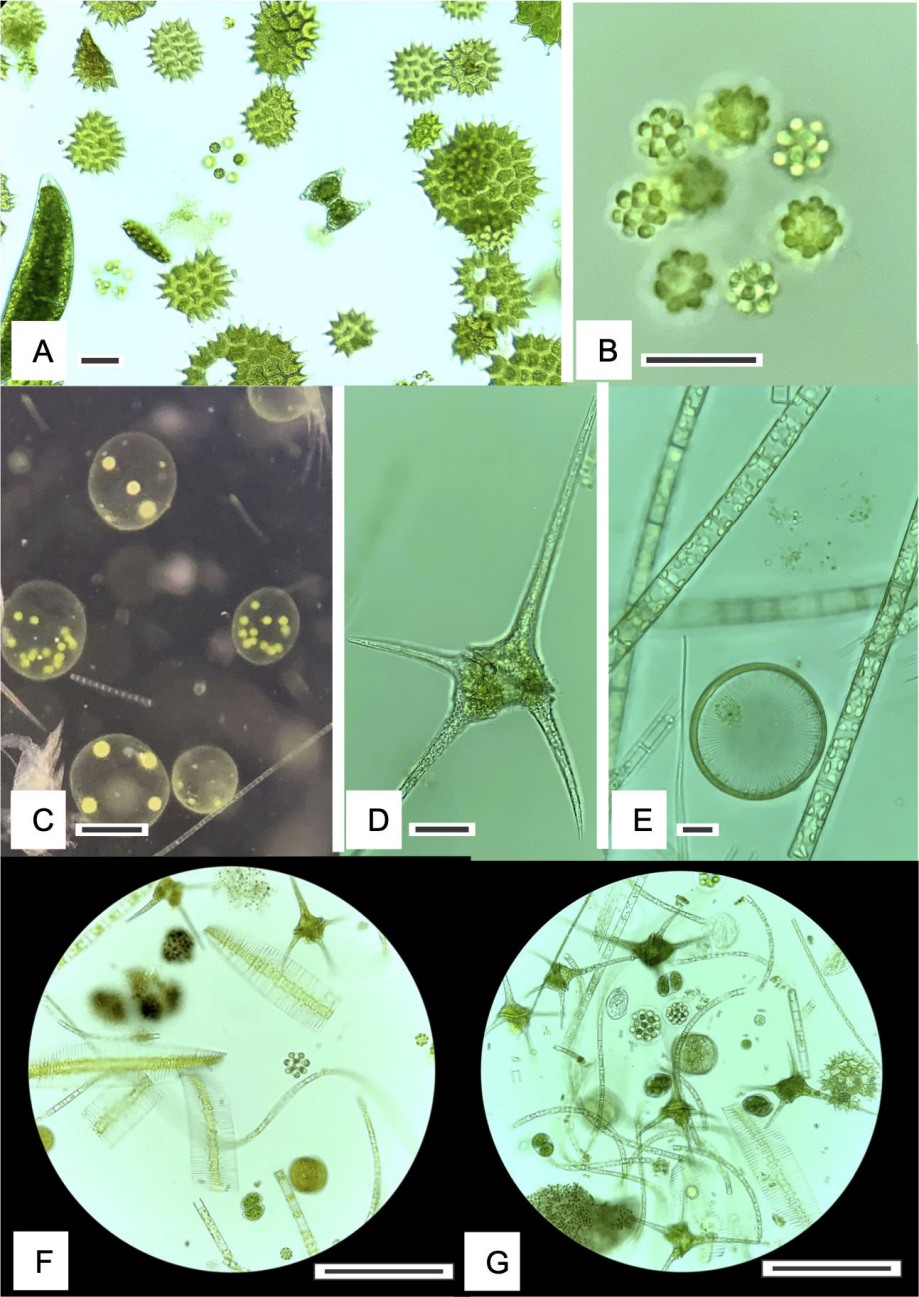
Protistan phytoplankton commonly observed in Clear Lake during the study period. (**A**) Chlorophyte (*Pediastrum*). (**B**) Colonial chlorophyte (unidentified). (**C**) Colonial chlorophyte (*Volvox*). (**D**) Dinoflagellate (*Ceratium*). (**E**) Diatoms (colonies and one solitary). (**F-G**) Mixed assemblages of phytoplankton observed in plankton tows (20 µm mesh) dominated by diatoms, dinoflagellates, and chlorophytes. One large *Microcystis* colony can be seen at lower left in (**G**). Marker bars are 20 µm (**A, B, D, E**) and 200 µm (**C, F, G**).

**Fig 7:**
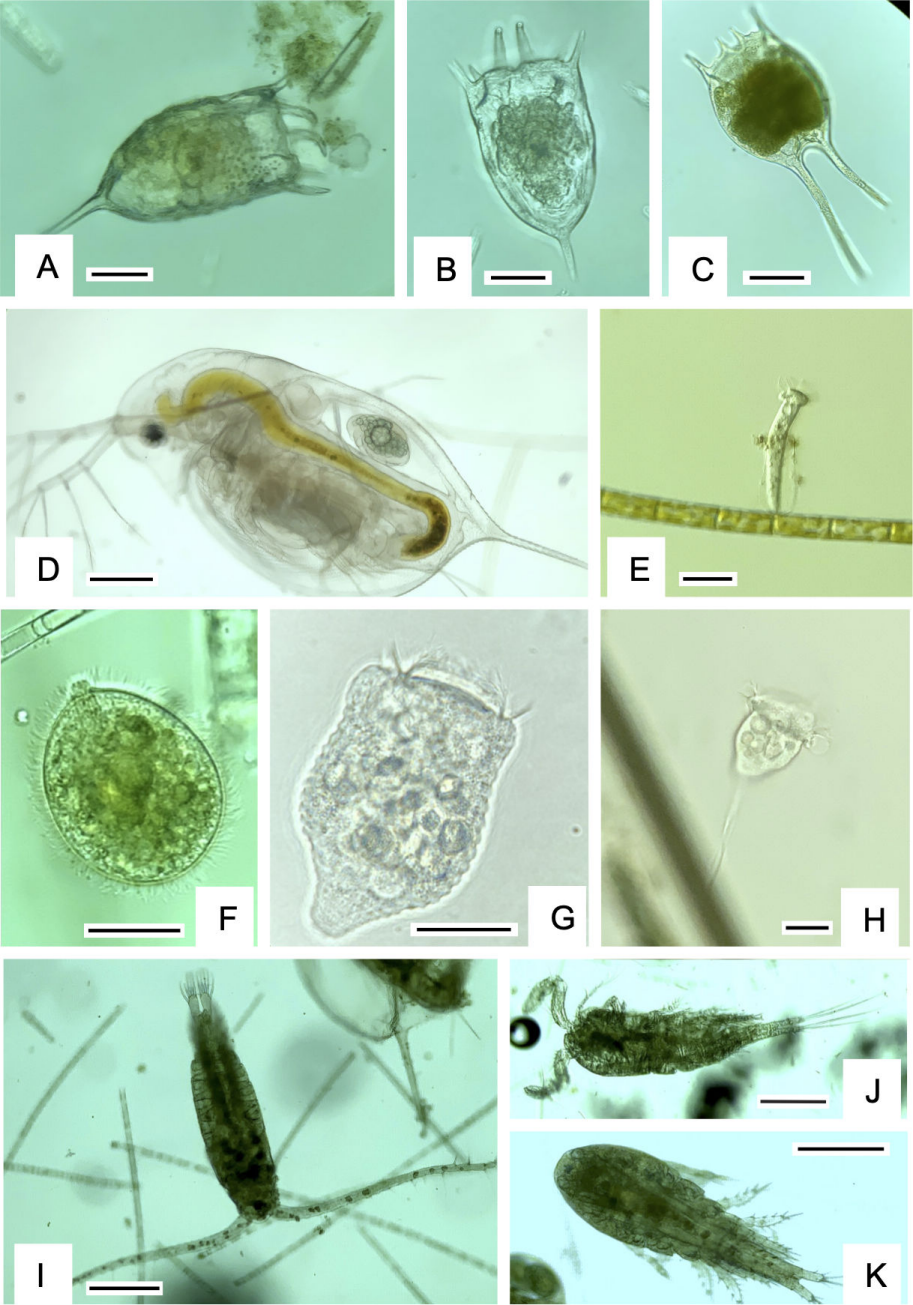
Heterotrophic protistan and metazoan taxa commonly observed in Clear Lake during the study. (**A-C**) Rotifer species. (**D**) Cladoran (*Daphnia*). (**E**) Loricate, attached peritrichous ciliate. (**F**) Large free-living ciliate. (**G, H**) Unattached (**G**) and attached (**H**) peritrichous ciliates. (**I–K**) Copepod species. Marker bars are 20 µm (**A–C, E–H**) and 200 µm (**D, I–K**).

PCoA also revealed seasonal differences in the eukaryotic assemblage composition during 2021 (Fig. 4B), although the separation between most months was not as distinct as for the bacterial assemblage (Fig. 4A). This seasonal trend was particularly evident from changes in the dominance of photosynthetic groups during summer and fall (Fig. 4D and 6). Chlorophytes were dominant during summer, whereas diatoms were more abundant during the fall months. Even within a particular group, we observed seasonal trends in the relative abundances of genera (Fig. S8A). Another algal group, the cryptophytes, were also significant members of the overall community, especially during August, September, and October. Heterotrophs, including ciliates, metazoans, and fungi, also made up significant proportions of the eukaryotic assemblage (Fig. 4D and 7). Ciliates were especially abundant and were present throughout summer and fall during 2021. Ciliate genera also changed throughout the two seasons, with species belonging to the Halteriidae family more abundant during the summer, whereas *Tintinnidium* strongly dominated in October (Fig. S8A). In contrast to the seasonality observed for some protistan taxa, metazoans appeared to have site-specific preferences. They were abundant in Oaks and Lower Arm sites throughout the two seasons, where the cyclopoid copepods dominated the community (Fig. 4D; Fig. S8A).

### Interannual and spatial differences in the microbial communities of Clear Lake

**Fig 8:**
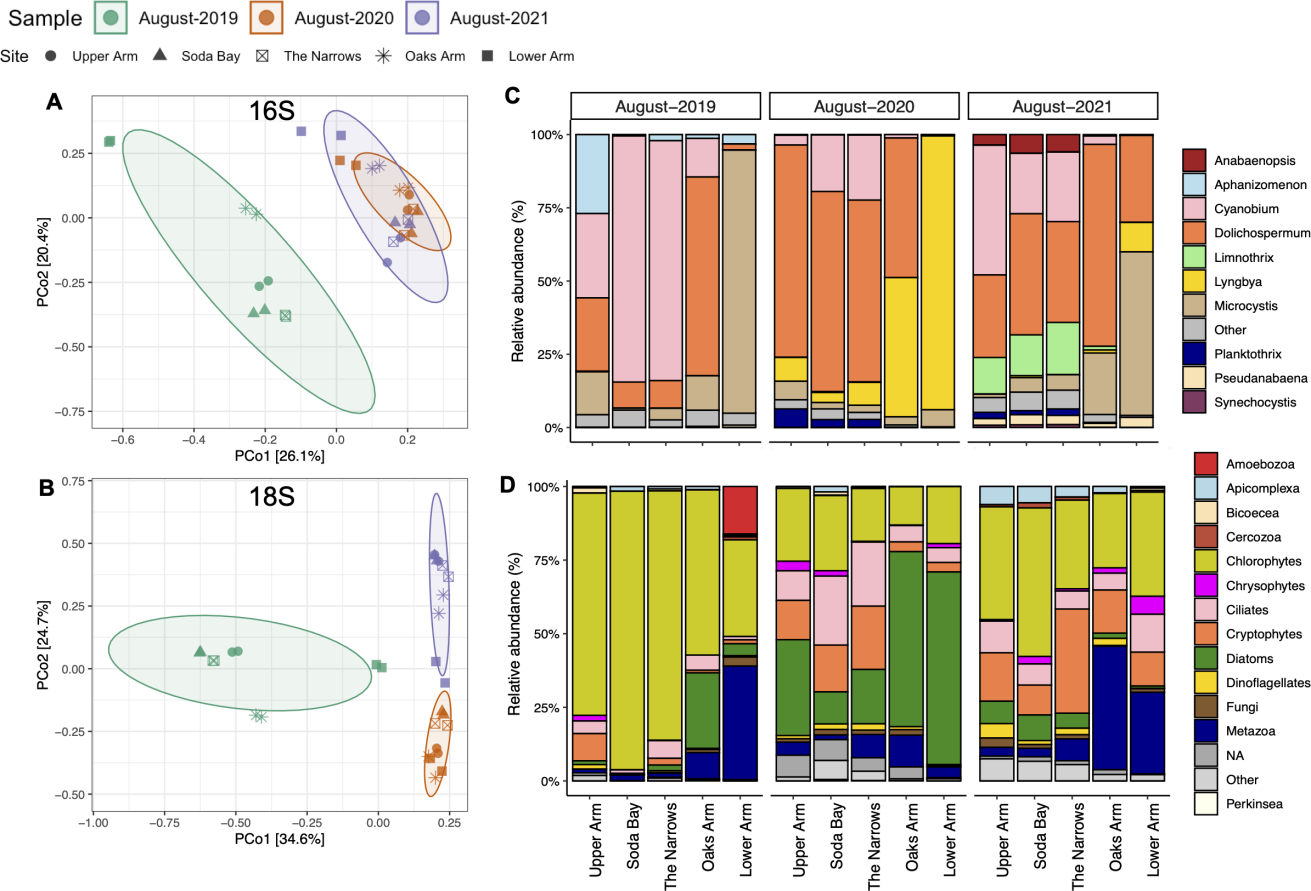
Interannual changes in bacterial and eukaryotic assemblage diversity and composition at different sites in Clear Lake during August for three successive years. PCoA ordination plots depicting Bray–Curtis dissimilarity of the bacterial (**A**) and eukaryotic (**B**) assemblage composition. Each point represents one sample or replicate. Sampling sites are depicted by different shapes, and the year of collection is represented with different colors (2019–green, 2020–orange, 2021–purple). Interannual changes in the relative abundance of major taxonomic groups of the cyanobacterial assemblage composition (**C**) at the genus level and eukaryotic assemblage composition (**D**) at the class or phylum level. Relative abundance data are averaged for the two replicates for each sample. The five sampling sites are shown on the *x*-axis.

Interannual and spatial differences in the microbial community composition were also evaluated (Fig. 8). PCoA indicated that the August 2019 bacterial assemblage was quite dissimilar to the 2020 and 2021 assemblages, while the latter two assemblages tended to cluster closely. Cyanobacterial sequences constituted a greater percentage of the bacterial reads in the 2019 samples relative to the other 2 years (Fig. 2D), and the cyanobacterial genus *Microcystis* constituted most of the cyanobacterial assemblage in Lower Arm during 2019, unlike the other sampling sites (Fig. 8C). A single ASV that best matched *Microcystis aeruginosa* NIES-90 was responsible for the *Microcystis* bloom in Lower Arm during August 2019. That *Microcystis* bloom also corresponded with the only site with detectable levels of microcystins in Clear Lake in August 2019 (Fig. 1B). Other sites were either dominated by species of the picocyanobacterium *Cyanobium* or the potentially toxic diazotrophic genus, *Dolichospermum* (Fig. 8C).

In contrast, during 2020, a different, potentially toxin-producing cyanobacterial genus, *Lyngbya*, dominated at the Lower Arm sampling site (Fig. 8C). *Lyngbya* and *Dolichospermum* also dominated at the Oaks Arm site in 2020. Like *Microcystis*, a single ASV was responsible for the *Lyngbya* bloom, which best matched *Lyngbya aestuarii* CCNP1324. At all the other sites in 2020, *Dolichospermum* and *Cyanobium* dominated the cyanobacterial assemblage. The highest microcystin concentrations were observed in August 2021 across all 3 years. That year, microcystins were not only detected at all sites, but the concentrations were approximately fourfold higher at the Lower Arm site compared with the other years (Fig. 1B). Several potential toxin-producing genera, including *Microcystis*, *Dolichospermum*, and *Lyngbya*, dominated Lower Arm in 2021 (Fig. 8C).

Unlike the bacterial assemblage, we observed clear interannual separation among the 18S communities for all 3 years, where samples for a particular year clustered near each other but away from other years (Fig. 8B). These trends were evident from examination of the taxonomic composition. The eukaryotic assemblage was strongly dominated by chlorophytes in 2019, while diatoms were more prevalent in 2020, and a mixture of chlorophytes, cryptophytes, and metazoa was common in 2021 (Fig. 8D).

### Influence of environmental factors on microbial community dynamics in Clear Lake

In the previous analysis, we observed strong seasonal and interannual patterns in the microbial diversity and community composition of Clear Lake, in addition to location-specific differences. Next, we attempted to identify which environmental variables might be contributing to those changes using PERMANOVA to evaluate the environmental factors that explain significant variance in the bacterial and eukaryotic assemblage diversity. The analysis of the interannual samples (August in 2019, 2020, and 2021) indicated that microcystin concentration, chlorophyll *a* concentration, total phosphorus, dissolved oxygen concentration, and conductivity explained annual variation in both the bacterial and eukaryotic assemblage (Table 1). Among those factors, the highest variance in the bacterial assemblage was explained by total phosphorus. In contrast, conductivity was the strongest predictor of the interannual trend for eukaryotes. For both the bacterial and eukaryotic microbial community, month-to-month variation was significantly explained by total phosphorus, temperature, microcystin concentration, and chlorophyll *a* (Table 2). Phosphorus concentration was particularly important as it explained ~30% of total month-to-month variance.

**TABLE 1:** PERMANOVA analysis of annual variation in bacterial (16S) and eukaryotic (18S) assemblages in Clear Lake^*a*^

Variable	R^2^ (16S)	*P*-value (16S)	R^2^ (18S)	*P*-value (18S)
Microcystin	0.111483*	0.01	0.094884*	0.002
Chlorophyll *a*	0.07795*	0.046	0.118288*	0.003
Total phosphorus	0.197426*	0.001	0.145678*	0.002
Total nitrogen	0.039763	0.222	0.023712	0.635
Temperature	0.059379	0.051	0.054115	0.135
Oxygen	0.107471*	0.001	0.128467*	0.002
Conductivity	0.124789*	0.002	0.186864*	0.001

^*a*^ The major independent environmental parameters are shown as variables. The strength of each variable contributing to variation in the beta-diversity (Bray–Curtis dissimilarity) are shown (R^2^). The significant factors contributing to overall variation are shown with an asterisk (*) with *P*-values below 0.05.

**TABLE 2:** PERMANOVA analysis of seasonal variation in bacterial (16S) and eukaryotic (18S) assemblages in Clear Lake^*a*^

Variable	R^2^ (16S)	*P*-value (16S)	R^2^ (18S)	*P*-value (18S)
Microcystin	0.14813057*	0.001	0.07753309*	0.001
Chlorophyll *a*	0.13363848*	0.001	0.13212728*	0.003
Total Phosphorus	0.30142935*	0.001	0.28803895*	0.001
Total Nitrogen	0.06382404*	0.014	0.04643814	0.180
Temperature	0.14963787*	0.001	0.13192243*	0.007
Oxygen	0.01203549	0.796	0.02318796	0.699
Conductivity	0.01669402	0.658	0.03391516	0.375

^*a*^ The major independent environmental parameters are shown as variables. The strength of each variable contributing to variation in the beta-diversity are shown (R^2^). The significant factors are shown with an asterisk (*) with *P*-values below 0.05.

**Fig 9:**
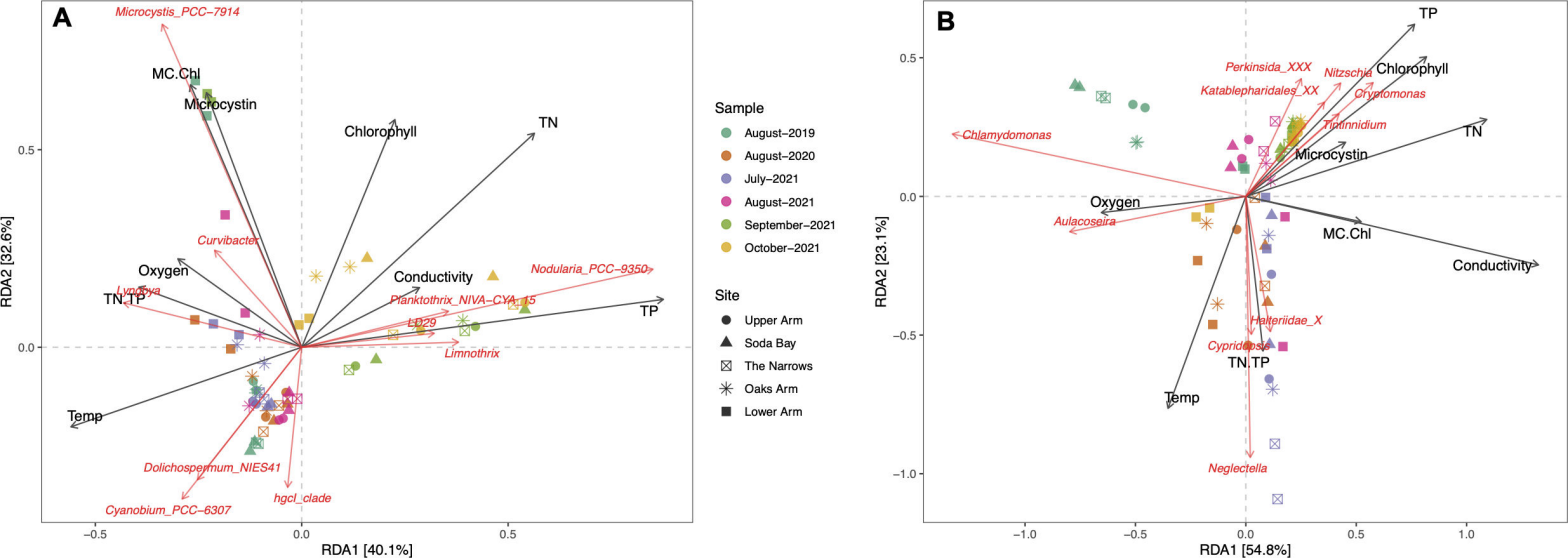
Redundancy analysis (RDA) of the top ten taxa in Clear Lake of the (A) bacterial assemblage and (B) eukaryotic assemblage at the genus level, constrained by the environmental variables measured for all samples (years and months). Variation in taxa are shown in red, and variations in environmental variables are shown in black. TN, total nitrogen; TP, total phosphorus; Temp, temperature; MC.Chl, microcystin:chlorophyll *a* ratio; TN.TP, TN:TP ratio.

**Fig 10:**
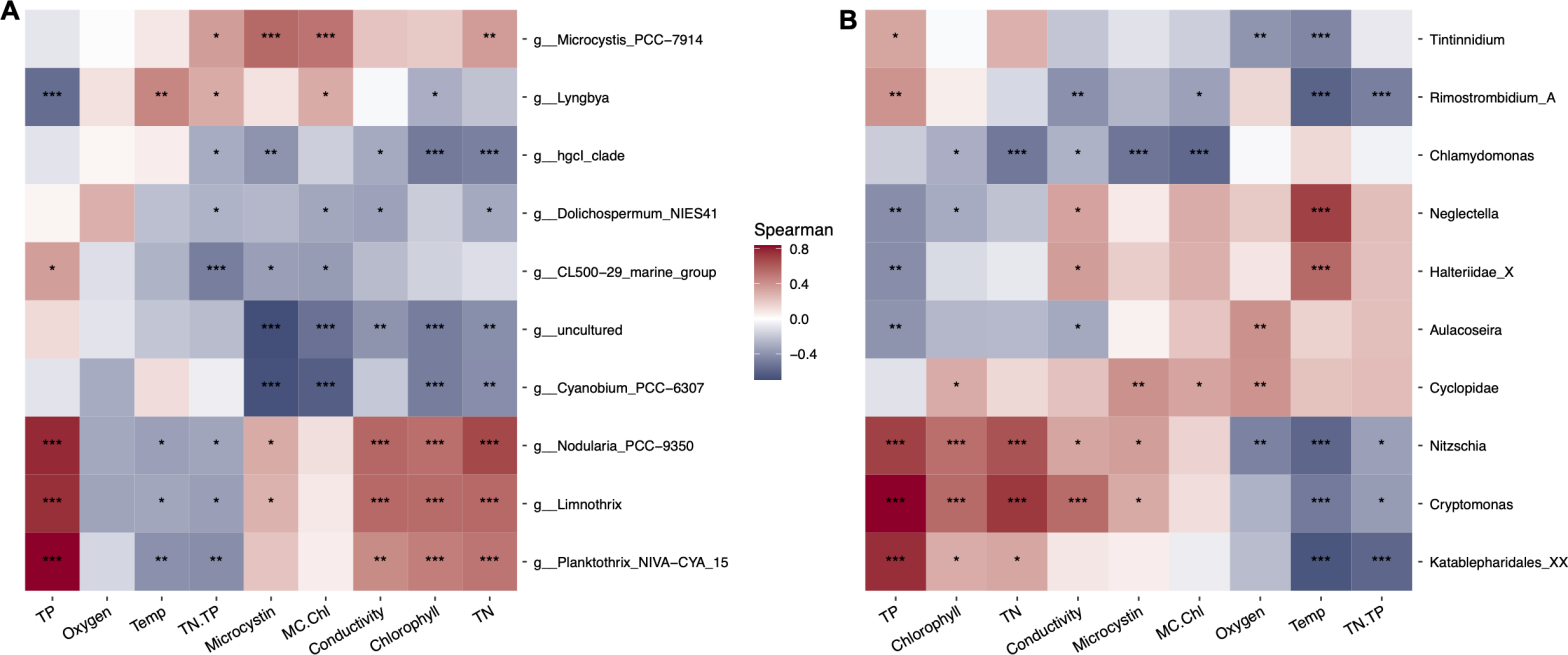
Spearman correlation heatmap of the top 10 cyanobacterial (**A**) and eukaryotic (**B**) genera and the environmental variables in Clear Lake for all the sampling dates. Positive correlations are shown in red, and negative correlations are shown in blue. Statistically significant *P*-values for correlations are shown with star(s) (*, **, ***) after adjustment with false discovery rate, with the number of stars representing increasing strength of significance (*P < 0.05, **P < 0.01, ***P < 0.001). TN, total nitrogen; TP, total phosphorus; Temp, temperature; MC.Chl, microcystin:chlorophyll *a* ratio; TN.TP, TN:TP ratio.

In addition to identifying the environmental factors that contribute to seasonal and annual trends in microbial diversity, genus-level redundancy analysis (RDA) and Spearman correlation analysis were conducted on the entire sample set to further understand the influence of environmental variables on specific taxa abundance trends and to identify the environmental triggers that can predict the presence of certain important bacterial and eukaryotic taxa in Clear Lake (Fig. 9 and 10). An RDA of the top 10 most abundant bacterial taxa from all samples showed neat division of summer (July, August) and fall (September, October) samples along the primary RDA axis, which explained over 40% of total variance (Fig. 9A). That variance was most significantly explained by temperature, total phosphorus, and conductivity. The second RDA axis explained 32% of variance and was constrained by microcystin and chlorophyll *a* concentration, which explained location-specific differences in the community. Both *Microcystis* and *Lyngbya* were significantly positively correlated with microcystin (*P* < 0.001) and microcystin:chl ratio (*P* < 0.05), respectively, as well as TN:TP ratio (*P* < 0.05) (Fig. 10A). Moreover, *Lyngbya* was also significantly negatively (*P* < 0.001) correlated with total phosphorus levels and showed a positive relationship with temperature (*P* < 0.01). Dominant summer taxa, such as *Cyanobium* and *Dolichospermum*, were negatively correlated with nitrogen (*P* < 0.01 and *P* < 0.05) and microcystin:chlorophyll *a* ratio (*P* < 0.001 and *P* < 0.05). In contrast, the dominant fall taxa *Anabaenopsis/Nodularia*, *Planktothrix,* and *Limnothrix* clustered together with the fall samples in the RDA plot and were influenced by similar season-specific environmental factors (Fig. 10A).

RDA of the top 10 eukaryotic genera for all samples revealed a more subtle separation between the samples, compared with the bacterial analyses (Fig. 9B). The primary RDA axis explained 54% of variance and was constrained by conductivity and dissolved oxygen concentration. Samples were divided according to year and season along the primary axis. On the other hand, the secondary RDA axis explained 23% of variance with temperature and TN:TP ratio being the dominant constraining factors. The two dominant chlorophyte genera, *Chlamydomonas* and *Neglectella*, were both negatively correlated with chlorophyll *a* levels in the lake (*P* < 0.05) (Fig. 10B). However, where on one hand *Chlamydomonas* was strongly negatively correlated with microcystin:chl levels (*P* < 0.001), *Neglectella* showed a weak positive correlation. Among diatoms, the two dominant taxa *Aulacoseira* and *Nitzschia* displayed contrasting dynamics with most environmental factors. Most notably, *Nitzschia* positively correlated, but *Aulacoseira* negatively correlated with total phosphorus (*P* < 0.001 and *P* < 0.01) and conductivity (*P* < 0.05 for both) (Fig. 10B). A dominant cryptophyte, *Cryptomonas* had similar correlation trends as *Nitzschia* and aggregated with that diatom on the RDA plot (Fig. 9B and 10B). Heterotrophic members of the eukaryote community, such as the ciliates *Tintinnidium* and *Rimostrombidium*, had a strong positive correlation with phosphorus (*P* < 0.05 and *P* < 0.01) and a negative relationship with temperature (*P* < 0.001) (Fig. 10B). In contrast, ciliates belonging to the Halteriidae family were negatively correlated with phosphorus (*P* < 0.01) and positively correlated with temperature (*P* < 0.001). Finally, a cyclopoid copepod showed a strong positive relationship with microcystin (*P* < 0.01) and oxygen (*P* < 0.01).

## DISCUSSION

Interactions between various members of the microbial community (cyanobacteria, heterotrophic bacteria, photosynthetic, mixotrophic or heterotrophic protists, and other microzooplankton) in an aquatic system are critical in defining food-web structure and dynamics as well as nutrient cycling. Therefore, it is not only important to understand cyanobacterial diversity and composition, but it is also essential to characterize the co-occurring microbes that closely interact with cyanobacterial members during cyanoHABs. Yet, relatively few studies have attempted to characterize both the prokaryotic and eukaryotic components of the planktonic community using genetic approaches.

In this study, we employed 16S and 18S ribosomal RNA gene sequencing to characterize the bacterial and microbial eukaryotic assemblage associated with peak cyanoHAB months in Clear Lake. To our knowledge, this is the first comprehensive study of both prokaryotic and eukaryotic members of the microbial community in Clear Lake. In addition to identifying the dominant microbial taxa and their temporal and spatial patterns, we also identified several environmental factors that correlated with bloom vs non-bloom conditions.

### Environmental and HAB setting for microbial community analysis

Clear Lake is a naturally eutrophic and polymictic lake with high standing stocks of phosphorus and nitrogen that support robust algal blooms (i.e., very high chlorophyll *a*; Fig. 1C). However, annual and monthly temporal changes in these nutrients and other physicochemical parameters contribute to substantial variations in the taxonomic composition and toxicity of these blooms (Fig. S1 and S2). During our study period, algal/cyanobacterial blooms of different magnitudes occurred in various Arms of the lake as we encountered both extremely toxic months as well as months when toxin levels were undetectable or below the California recreational trigger levels (Fig. 1B, 5A and B). Highest microcystin concentrations were recorded in September 2021, consistent with the trends observed in the historic analysis of toxin data, which showed that highest toxin concentrations have generally been observed during August and September (3). Overall, 2021 was a particularly toxic year with significantly higher microcystin and chlorophyll levels compared with August 2019 and August 2020 (Fig. 1B and C). Interannual variations in chlorophyll and microcystin concentrations are frequent occurrences in Clear Lake, and substantial differences in the year-to-year maxima of these parameters were reported in the recent review of Clear Lake historical data (3). Annual fluctuations in temperature, precipitation, lake discharge, and evaporation, as well as release of nutrients from sediments have been proposed to be major contributors to these interannual differences. In many eutrophic lakes around the world, N, P, or both N and P can play important roles in promoting cyanobacterial blooms (29–32). During our study, high concentrations of both total N and total P were observed in 2021, which may have been driving the higher frequency and density of toxic cyanobacterial blooms that year (Fig. S1 and S2). However, overall, the total N and P concentrations recorded during our study period are within the range of values generally observed at Clear Lake (3).

Spatial disparity in bloom intensity and community composition among the three Arms of Clear Lake as observed in this study are well known (1, 3, 7) and complicate the ability to predict lake-wide toxic conditions from parameters that are readily measured, such as chlorophyll *a* concentration. Overall, microcystins and chlorophyll *a* concentrations were weakly correlated across all sampling sites and dates (Fig. S3), and consequently chlorophyll *a* concentrations have sometimes been proposed as a sentinel for toxin monitoring (3, 33, 34). There was considerable variability in this relationship, however, as some samples with relatively high chlorophyll *a* concentration had relatively low concentrations of microcystins (e.g. Oaks Arm in August 2021 and Upper Arm, Soda Bay, and The Narrows in September 2021; Fig. 1B and C). Such blooms were variously dominated by chlorophytes, diatoms, or species of cyanobacteria that were not producing toxins. These observations describe a microbial community that is often abundant but spatially and temporally dynamic in composition and toxin status.

### Core communities in Clear Lake were dominated by photosynthetic taxa

Our multi-domain molecular survey revealed that the Clear Lake bacterial assemblage was dominated by cyanobacteria. The dominant heterotrophic bacterial groups were the Proteobacteria, Bacteroidota, and Actinobacteria (Fig. 2), which have also been observed to be commonly associated with cyanobacterial blooms in other eutrophic lakes (33, 35–38), and may indicate as-yet undocumented metabolic or trophic relationships with the cyanobacteria. Microeukaryotes co-occurring during cyanobacterial blooms were also dominated by photosynthetic groups, such as chlorophytes, diatoms, and cryptophytes, as well as significant contributions of heterotrophic groups, such as ciliates and metazoans (Fig. 3). These high abundances of both bacterial and eukaryotic photosynthetic groups agree with the consistently high chlorophyll *a* levels and primary productivity observed in Clear Lake.

### The picocyanobacterium *Cyanobium* is an important member of the Clear Lake cyanobacterial assemblage

The picoplanktonic cyanobacterium *Cyanobium,* which has been completely missed by microscopic surveys due to its small size, was a significant member of the cyanobacterial assemblage in the western part of Clear Lake during our study (Fig. 4C and 8C). *Cyanobium* was particularly abundant during summer months (July and August) and generally in regions within the lake (Upper Arm) that correlated with “non-bloom” conditions (Fig. 1C, 4C and 8C; Table S2). There have been recent reports of this picocyanobacterium co-occurring prior to proliferation of cyanoHABs in other eutrophic lakes, where it was also abundant during summer and in absence of blooms (35, 39, 40). The closely related genus *Synechococcus* has been identified once before in Clear Lake, also in a molecular survey (9), emphasizing the importance of molecular tools for characterizing the full breadth of the microbial community. Both *Synechococcus* and *Cyanobium* are important and ubiquitous members of the cyanobacterial assemblage in freshwater systems (41, 42). *Synechococcus* has been shown to be especially competitive in oligotrophic environments (42), in offshore Lake Erie where *Microcystis* abundance was lower (36), and during low nutrient conditions of summer months due to its adaptation to warm waters and its ability to quickly take up nutrients (43, 44). Some studies have also shown that picocyanobacteria, such as *Synechocystis* and *Synechococcus*, can produce multiple cyanotoxins, including microcystins (45). Moreover, there is evidence of at least one *Cyanobium* species (*Cyanobium rubescens* SAG 381) that is capable of microcystin production (46). However, we found that *Cyanobium* abundance was significantly negatively correlated with microcystin levels (Fig. 10A) and thus unlikely to have been a contributor to the toxin during our study. Concurrently, Clear Lake *Cyanobium* was also negatively correlated with both nitrogen and phosphorus and chlorophyll concentrations, indicating that it may experience a competitive advantage during periods of low nutrients and non-bloom conditions.

In contrast to the unexpected presence of *Cyanobium*, the cyanobacterium *Gloeotrichia* was readily observed by microscopy and the naked eye at specific times and locations during our study (Fig. 5G and H; Table S3) but was never observed in our sequencing data set. Dense blooms of diazotrophic *Gloeotrichia* have been frequently documented by microscopy in Clear Lake (3), especially during early summer when nitrogen levels are relatively low. During our study, our microscopic survey showed that *Gloeotrichia* was particularly abundant during August 2019 and was also observed in July 2021 (Table S3); however, we were unable to detect the taxon in our amplicon survey of those dates (Fig. 4C and 8C). The colonial *Gloeotrichia* cells are notoriously difficult to disrupt due to the dense mucilaginous matrix that surrounds the filamentous body (47, 48). Several procedures were conducted, to no avail, to remedy this problem (data not shown). We conclude that our sample processing methods were ineffective in accessing *Gloeotrichia* nucleic acids, despite their prevalence in some samples. The contrasting results provided from different methodologies (DNA sequencing vs microscopy) highlight the importance of using multiple approaches for cyanoHAB surveys.

### Clear Lake cyanobacterial assemblage exhibited seasonal succession of diazotrophic and non-diazotrophic bloom-causing taxa

Diazotrophic cyanobacteria are an important component of the Clear Lake bacterial assemblage as they may be responsible for a major source of nitrogen for the lake, in turn promoting proliferation of other non-diazotrophic cyanobacteria and eukaryotes (5). In agreement with that generalization, we observed a temporal succession of different nitrogen-fixing and non-nitrogen-fixing cyanobacteria in our seasonal study. The non-nitrogen-fixing *Microcystis* blooms appeared to follow the increase in total nitrogen in the lake, presumably in part due to the diazotroph blooms earlier or concomitantly in the year, a characteristic pattern of *Microcystis* blooms (49–51). A significant positive correlation between *Microcystis* abundance and total nitrogen (but not phosphorus) was observed in our Spearman correlation analyses of all samples (Fig. 10A). Other recent studies have noted that nitrogen may be equally, if not more important than phosphorus for proliferation of toxic *Microcystis* blooms (52).

TN:TP values in the lake appear to substantiate this scenario of dominance of diazotrophic cyanobacteria followed by non-diazotrophs. TN:TP values were highest during July 2021, a finding potentially indicating an impact of the diazotrophic cyanobacterium *Gloeotrichia* in early-season nitrogen loading of the lake (Fig. S1H) (3). During August, there was a significant increase in phosphorus levels and concomitant decrease in TN:TP (Fig. S1H). TN:TP ratios decreased from July through October 2021 in the lake, despite substantial increases in both TN and TP over the same time period, presumably explaining the appearance and dominance of another filamentous diazotrophic genus at that time, *Nodularia*/*Anabaenopsis* (Fig. 4; Fig. S1 and S7).

Non-nitrogen-fixing filamentous taxa, such as *Limnothrix* and *Planktothrix*, were also abundant during September and October, respectively (Fig. 4C; Fig. S1 and S7). In addition to *Microcystis*, *Planktothrix* has also been shown to be stimulated following nitrogen loading events (53) and may have proliferated in October due to the increase in TN following the *Nodularia* bloom. *Nodularia*, *Planktothrix,* and *Limnothrix* have also been previously observed to co-occur, for example, in a winter bloom in a temperate eutrophic lake (54). Moreover, these three taxa were significantly positively correlated with total phosphorus and nitrogen in our study (Fig. 10A). Overall, there appears to be a distinct seasonal succession of potentially toxin-producing diazotrophic and non-diazotrophic cyanobacterial blooms during summer and fall seasons in Clear Lake.

### Phosphorus still plays a major role in shaping the overall microbial community composition

Stark monthly changes in the microbial community composition were observed during our 2021 summer and fall sampling period, with a distinct seasonal succession within the cyanobacterial and microeukaryotic assemblage (Fig. 4 and 9A; Fig. S7 and S8A). Phosphorus levels have been generally implicated in determining the cyanobacterial assemblage structure in Clear Lake, and measures have been taken to reduce the total phosphorus loads as noted above (3). In our study, we observed that the phosphorus load in Clear Lake increased during fall, significantly changing every month (Fig. S1D). PERMANOVA and RDA analyses showed that phosphorus was the most important variable in explaining the monthly/seasonal variance in both bacterial and eukaryotic assemblage composition, followed by temperature (Table 2; Fig. 9A). These monthly differences were especially apparent in the succession of different cyanobacterial taxa as noted above (Fig. 4C; Fig. S7). Recent studies have also shown that internal phosphorus loading is a major source of total phosphorus in Clear Lake and helps in proliferation of nitrogen-fixing cyanobacteria (55).

### The co-occurring bacterial and eukaryotic assemblages also exhibit seasonal trends

Cyanobacterial taxa continuously interact with both the abiotic and biotic components of their environment. The co-occurring bacterial taxa play an important role in the progression of cyanobacterial blooms by regulating nutrient limitation through the production, utilization, or remineralization of organic compounds (56). Conversely, cyanobacterial bloom intensities have been shown to alter community composition and diversity of co-occurring bacterial and eukaryotic communities (14, 57, 58). Within the bacterial assemblage of Clear Lake, the dominant actinobacteria from hgcI clade (also known as acI) and CL500-29 exhibited opposite seasonal trends (Fig. S7). Whereas the hgcI clade had higher relative abundances during summer and lower abundances during fall, the CL500-29 marine group had higher relative abundances during fall compared with summer. Both these genera are prevalent in eutrophic waters where they are proficient degraders of dissolved organic carbon compounds (59, 60) and have also been shown to co-occur as dominant bacterial genera of cyanobacterial blooms (61, 62). Shifts in the relative abundances of these two bacterial genera occurred during the summer and fall of 2021 in Clear Lake when different cyanobacteria dominated the phytoplankton community (Fig. 4C; Fig. S7). We speculate that the inverse temporal relationship between these two actinobacterial genera reflects an association with blooms formed by different cyanobacterial species. The directionality of these relationships is yet to be determined. That is, it is presently unknown if these heterotrophic bacterial genera somehow conditioned the water for the success of specific cyanobacteria, or if specific cyanobacteria somehow favor the success of certain heterotrophic bacteria. Understanding these biotic interactions will provide new insight into the factors structuring microbial communities.

The eukaryotic assemblage in Clear Lake also showed monthly succession during 2021; however, a slower succession of the eukaryotes was observed compared with the bacterial assemblage (Fig. 4B and D). This finding is not surprising given the different generation times of prokaryotic and eukaryotic species. The eukaryotic seasonal succession was predominantly a result of changes in relative abundance of chlorophytes, diatoms, and cryptophytes (Fig. 4D). As noted above, chlorophytes were relatively more abundant during the summer months, and diatoms were more abundant during the fall. Moreover, within a specific group, genus level differences were also evident. For example, among the chlorophytes, *Neglectella* exhibited greater abundances during July, whereas *Chlamydomonas* was more prevalent during August (Fig. 4D; Fig. S8A). Among diatoms, *Aulacoseira* was relatively more abundant during July; however, *Nitzschia* dominated the following months. Somewhat surprisingly, few eukaryotic taxa correlated negatively with microcystin concentrations (Fig. 10B). Our expectation was that the presence of microcystins might negatively impact sensitive eukaryotic taxa, as some taxa belonging to dinoflagellates and green algae have been previously reported to be negatively affected by these toxins (63). However, only *Chlamydomonas* and the ciliate *Tintinnidium* exhibited a strong and a weak negative correlation, respectively, with microcystins. *Chlamydomonas* species have been specifically shown to be quite sensitive to microcystins in laboratory studies (64, 65) and we propose that this algal genus is most strongly affected by the microcystin concentrations in Clear Lake.

### Clear Lake microbial community displays strong interannual variability

Our multi-year analysis of the microbial community during August in Clear Lake showed that both the bacterial and eukaryotic assemblage varied interannually. Although the contributions of major phyla remained relatively similar across the two domains (Fig. 2D and 3D), genus-specific differences were still pronounced (Fig. S8B and S9). Within the bacterial assemblage, specific differences in the cyanobacterial assemblage appeared to drive the annual differences, whereas the heterotrophic bacteria displayed somewhat similar relative abundances across the years (Fig. S9). Similar annual trends were also observed among the co-occurring microeukaryotes, where annual changes were predominantly driven by photosynthetic groups, such as chlorophytes and diatoms (Fig. 8D). For example, *Chlamydomonas* dominated the eukaryotic assemblage during August 2019, whereas in August 2020, the other dominant chlorophyte *Neglectella* and the diatom *Aulacoseira* were more prevalent (Fig. S8B).

### Spatial trends in the cyanobacterial assemblage influence the co-occurring eukaryotic assemblage and correspond to microcystin distribution in Clear Lake

Spatial trends in the overall bacterial and eukaryotic assemblages were evident from PCoAs and occurrence of specific taxa at various sites (Fig. 4A and B, 8A and B). Although strong temporal trends were observed for Clear Lake sites on the western part of the lake, Oaks Arm and Lower Arm sites tended to cluster closer to each other across various months and years. These trends indicate a greater homogeneity in the composition of microbial taxa at Lower Arm and Oaks Arm compared with the other three sites. This spatial abundance pattern was most significantly observed in the distribution of *Microcystis*, which was particularly concentrated at the Lower Arm and Oaks Arm sampling sites (Fig. 4C and 8C). Moreover, among all the potential toxin-producing genera, *Microcystis* showed the strongest positive relationship with microcystin concentration (Fig. 9A and 10A). Regular monitoring of microcystin levels in Clear Lake has been consistently and significantly higher in Oaks and Lower Arm relative to Upper Arm (https://www.bvrancheria.com/clearlakecyanotoxins) and have generally coincided with higher abundances of *Microcystis*. Similarly, *Lyngbya* was also more prevalent at Oaks and Lower Arm sites but did not correlate strongly with microcystins (Fig. 10A). Predominant wind direction across the lake (northwest-to-southeast) likely plays an important role in the accumulation of these surface-associated cyanobacterial taxa at the eastern end of Clear Lake.

These disparate cyanobacterial blooms presumably influenced the composition of the eukaryotic assemblage as well. For example, *Microcystis* dominated the Lower arm sites during 2019 and 2021, whereas *Lyngbya* dominated Lower Arm and Oaks Arm in 2020 (Fig. 8C). Concurrently, metazoans (mainly cyclopoid copepods) were specifically prevalent at Lower Arm and Oaks Arm in 2019 and 2021 but not during 2020 when *Lyngbya* dominated (Fig. 8D). The periods of higher abundances of copepods also overlapped with lower relative abundances of chlorophytes. We speculate that copepods may have experienced a selective advantage at these sites if they were able to either prey on the nutritious chlorophytes (66) or perhaps directly on *Microcystis* (16). Furthermore, some mixotrophic chrysophytes have been shown to be efficient grazers of toxic cyanobacteria, and biotic interactions among them have been frequently observed (67–69). More specifically, toxin production by *Microcystis aeruginosa* has been shown to enhance cyanobacterivory in *Cryptomonas* (70). Interestingly, this mixotrophic flagellate was more abundant during the fall, especially at the Lower Arm site where *Microcystis* abundance was greatest and also positively correlated with microcystin levels (Fig. 10B). Among the diatoms, *Nitzschia* was particularly abundant during September 2021 when the highest microcystin concentrations were observed. Apart from cyclopoid copepods and *Cryptomonas*, *Nitzschia* was the only other microeukaryote that positively correlated strongly with microcystin concentration (Fig. 10B). Unlike *Chlamydomonas*, which has been shown to compete with *Microcystis* for resources (71), *Nitzschia* cells have been found to be physically associated with *Microcystis aeruginosa* colonies (72). Consequently, we speculate that the spatial distributions of specific cyanobacterial genera may influence the eukaryotic assemblage distribution through physical and allelopathic interactions.

## Data Availability

Raw sequence data associated with this project are available on NCBI under the accession number PRJNA1206199. The R code associated with sequence filtering, normalization, and plotting is available on GitHub (https://github.com/IKalra889/Clear-Lake-amplicon-manuscript).

## SUPPLEMENTAL MATERIAL

Supplemental material aem.00011-25-s0001.docx Tables S1 to S3; Figures S1 to S9.

ASM does not own the copyrights to Supplemental Material that may be linked to, or accessed through, an article. The authors have granted ASM a non-exclusive, world-wide license to publish the Supplemental Material files. Please contact the corresponding author directly for reuse.
