# Supplementary material for: Temporal and spatial dynamics of harmful algal bloom-associated microbial communities in eutrophic Clear Lake, California
Source: Appl Environ Microbiol. 2025 Mar 28;91(4):e00011-25. doi: 10.1128/aem.00011-25 (PMC12016506; doi:10.1128/aem.00011-25)
Supplement: Supplemental material — Tables S1 to S3; Figures S1 to S9. [file aem.00011-25-s0001.docx]

**SUPPLEMENTARY INFORMATION**

Temporal and spatial dynamics of harmful algal bloom-associated microbial communities

in eutrophic Clear Lake, California

Isha Kalra^a, #^, Brittany P. Stewart^a^, Kyra M. Florea^a^, Jayme Smith^b^, Eric A. Webb^a^, David A. Caron^a^

**SUPPLEMENTARY TABLES**

**Supp. Table S1:** Total number of 16S and 18S rRNA reads and ASVs before and after filtering for contaminants.

|  | **Raw Reads** | | **After Decontamination** | |
| --- | --- | --- | --- | --- |
|  | **16S** | **18S** | **16S** | **18S** |
| **Sequence reads** | 10,908,910 | 347,748 | 10,853,875 | 346,868 |
| **ASVs** | 8728 | 1265 | 8715 | 1265 |

**Supp. Table S2**: Sample dates and sites that belong to “bloom” vs “non-bloom” category based on Clear Lake Total Maximum Daily Loads (TMDL Chl *a* – 73 ug/L) threshold during the three-year sampling survey.

| **Sample Date** | **Bloom Site (Chl *a* >= 73 ug/L)** | **Non-Bloom Site (Chl *a* < 73 ug/L)** |
| --- | --- | --- |
| August 2019 | Soda Bay | Upper Arm |
|  | Lower Arm | The Narrows |
|  |  | Oaks Arm |
| August 2020 | Soda Bay | Upper Arm |
|  |  | The Narrows |
|  |  | Oaks Arm |
|  |  | Lower Arm |
| July 2021 | Oaks Arm | Upper Arm |
|  | Lower Arm | Soda Bay |
|  |  | The Narrows |
| August 2021 | Upper Arm |  |
|  | Soda Bay |  |
|  | The Narrows |  |
|  | Oaks Arm |  |
|  | Lower Arm |  |
| September 2021 | Upper Arm |  |
|  | Soda Bay |  |
|  | The Narrows |  |
|  | Oaks Arm |  |
|  | Lower Arm |  |
| October 2021 | Upper Arm |  |
|  | Soda Bay |  |
|  | The Narrows |  |
|  | Oaks Arm |  |
|  | Lower Arm |  |

**Supp. Table S3:** Microscopic relative abundance estimates of major phytoplankton taxonomic groups at Clear Lake. Minimum and maximum range of % relative abundance of each group is shown for each site within a sampling date.

| **Sample Date** | **Site** | **Microcystis** | **Dolicho-spermum** | **Gloeo-trichia** | **Lyngbya** | **Diatom** | **Green**  **algae** |
| --- | --- | --- | --- | --- | --- | --- | --- |
| **August 2019** | Upper Arm | 1-9 % |  | > 50 % |  | 1-9 % | 1-9 % |
|  | Soda Bay | N/A | N/A | N/A | N/A | N/A | N/A |
|  | The Narrows | > 50 % |  | < 1% |  | 1-9 % | 1-9 % |
|  | Oaks Arm | 1-9 % | - | > 50 % | - | 1-9 % | 1-9 % |
|  | Lower Arm | N/A | N/A | N/A | N/A | N/A | N/A |
| **August 2020** | Upper Arm | 1-9 % | 25-49 % |  | 10-24 % | 25-49 % | 1-9 % |
|  | Soda Bay | 1-9 % | > 50 % |  | 1-9 % | 10-24 % | 1-9 % |
|  | The Narrows | 1-9 % | 25-49 % |  | 10-24 % | 25-49 % | 1-9 % |
|  | Oaks Arm | 1-9 % | 10-24 % |  | 10-24 % | > 50 % | 1-9 % |
|  | Lower Arm | 1-9 % | < 1% |  | > 50 % | 25-49 % | < 1% |
| **July 2021** | Upper Arm | 1-9 % | < 1% |  | 25-49 % | 1-9 % | 25-49 % |
|  | Soda Bay | 1-9 % | < 1% | 1-9 % | 25-49 % | 1-9 % | 25-49 % |
|  | The Narrows | < 1% | < 1% |  | 1-9 % | 1-9 % | > 50 % |
|  | Oaks Arm | 1-9 % |  |  | > 50 % | 1-9 % | 10-24 % |
|  | Lower Arm | 10-24 % |  | 1-9 % | > 50 % | 1-9 % | 10-24 % |
| **August 2021** | Upper Arm | 1-9 % | 25-49 % |  |  |  | 10-24 % |
|  | Soda Bay | 10-24 % | > 50 % |  |  | 1-9 % | 10-24 % |
|  | The Narrows | 1-9 % | > 50 % |  |  | < 1% | 1-9 % |
|  | Oaks Arm | 1-9 % | > 50 % |  |  | < 1% | 1-9 % |
|  | Lower Arm | 25-49 % | 25-49 % |  | 10-24 % |  | 1-9 % |
| **September 2021** | Upper Arm | 1-9 % | > 50 % |  |  | < 1% | 1-9 % |
|  | Soda Bay | 1-9 % | 25-49 % |  |  | 1-9 % | 10-24 % |
|  | The Narrows | 1-9 % | 25-49 % |  |  | < 1% | 1-9 % |
|  | Oaks Arm | N/A | N/A | N/A | N/A | N/A | N/A |
|  | Lower Arm | > 50 % | 1-9 % |  | 1-9 % | 1-9 % | 1-9 % |
| **October 2021** | Upper Arm | 1-9 % | > 50 % |  |  | 1-9 % |  |
|  | Soda Bay | > 50 % | 10-24 % |  |  | 10-24 % | < 1% |
|  | The Narrows | 10-24 % | > 50 % |  |  | 10-24 % | 1-9 % |
|  | Oaks Arm | N/A | N/A | N/A | N/A | N/A | N/A |
|  | Lower Arm | 25-49 % |  |  |  | > 50 % |  |

**SUPPLEMENTARY FIGURES**

**Supp. Fig. S1.** Monthly differences in the environmental parameters in Clear Lake during summer and fall 2021. In the boxplots, horizontal lines represent medians, hinges represent the interquartile ranges (25% - 75%) and whiskers extend to 1.5xIQR. The points that fall outside the 1.5xIQR are shown as outliers. Data from all five sites for shown for each sampling date. Each point represents value obtained for a particular site/replicate at the specific sampling date. Statistically significant differences among means of different dates were calculated using anova test and are represented by different letters (e.g. a, b, etc.). TN: total nitrogen, TP: total phosphorus, MC:Chl: microcystin:chlorophyll *a* ratio.

**Supp. Fig. S2**. Interannual differences in environmental parameters during August in Clear Lake for three successive years (2019-2021). In the boxplots, horizontal lines represent medians, hinges represent the interquartile ranges (25% - 75%) and whiskers extend to 1.5xIQR. The points that fall outside 1.5xIQR are shown as outliers. Data from all five sites for shown for each sampling date. Each point represents value obtained for a particular site/replicate at the specific sampling date. Statistically significant differences among means of different dates were calculated using anova test and are represented by different letters (e.g. a, b, etc.). TN: total nitrogen, TP: total phosphorus, Temp: temperature, MC:Chl: microcystin:chlorophyll *a* ratio.

**Supp. Fig. S3.** Correlation between microcystin and chlorophyll *a* concentrations in Clear Lake during the 3-year sampling period (all sampling dates). The linear regression fit line and correlation coefficient are indicated in black in the plot along with the 95% confidence interval, which is shown with dashed line.

**Supp. Fig. S4**. Site-specific microbial composition at Clear Lake during summer and fall of 2021. Relative abundance of different bacterial phyla (A) and eukaryotic supergroup (B) are shown.

**Supp. Fig. S5**. Site-specific microbial composition at Clear Lake during August 2019, 2020 and 2021. Relative abundance of different bacterial phyla (A) and eukaryotic supergroup (B) are shown.

**Supp. Fig. S6.** Shannon diversity indices of the microbial community in Clear Lake. A. Jitter boxplot representing Shannon index for the bacterial assemblage averaged for all sites and replicates for the sampling month or year. B. Jitter boxplot representing Shannon index for the eukaryotic assemblage averaged for all sites and replicates for the sampling month or year. In the boxplot, horizontal lines represent medians, hinges represent the interquartile ranges (25% - 75%) and whiskers extend to 1.5xIQR. The points that fall outside the 1.5xIQR are shown as outliers. Statistically significant values are represented by different letters after anova test.

**Supp. Fig S7.** Heatmap showing monthly changes in the relative abundance of the top 22 most abundant Clear Lake bacterial genera from samples collected during summer and fall 2021. Individual rectangles represent the different sampling sites and replicates.

**Supp. Fig. S8**. Boxplots showing monthly (A) and interannual (B) changes in the relative abundance of the top 10 most abundant eukaryotic genera in Clear Lake. Horizontal lines represent medians, hinges represent interquartile ranges (25% - 75%) and whiskers extend to 1.5xIQR. The points that fall outside the 1.5xIQR are shown as outliers. Replicates and sites within a specific sampling date have been averaged.

**Supp. Fig. S9.** Heatmap showing interannual changes in the relative abundance of the top 22 most abundant Clear Lake bacterial genera from samples collected during August of three successive years (2019, 2020 and 2021). Individual rectangles represent the different sampling sites and replicates.
